# Variety of Antimicrobial Resistances and Virulence Factors in *Staphylococcus aureus* Isolates from Meat Products Legally and Illegally Introduced to Germany

**DOI:** 10.1371/journal.pone.0167864

**Published:** 2016-12-09

**Authors:** Anja Müller, Diana Seinige, Wiebke Jansen, Günter Klein, Ralf Ehricht, Stefan Monecke, Corinna Kehrenberg

**Affiliations:** 1 Institute of Food Quality and Food Safety, University of Veterinary Medicine Hannover, Foundation, Hannover, Germany; 2 Integrated Veterinary Research Unit, University of Namur, Namur, Belgium; 3 Alere Technologies GmbH, Jena, Germany; 4 InfectoGnostics research campus, Jena, Germany; 5 Institute for Medical Microbiology and Hygiene, Technische Universität Dresden, Dresden, Germany; Amphia Ziekenhuis, NETHERLANDS

## Abstract

Food products of animal origin can serve as a vehicle for *Staphylococcus* (*S*.) *aureus*, a facultative pathogen involved in a variety of diseases. As a result, international trade and illegal transportation of foodstuffs can facilitate the distribution of *S*. *aureus* over long distances. In this study, we investigated *S*. *aureus* isolates recovered from meat products confiscated from passengers returning from non-EU countries at two German airports and from samples of legally imported meats from non-EU countries. The aim was to characterize isolates in regard to their genetic relatedness as well as their antimicrobial resistance profiles and major virulence factors in order to assess potential risks associated with these products. The isolates were characterized by *spa* typing, MLST, macrorestriction analysis, microarray analysis and antimicrobial susceptibility testing. MRSA isolates were further characterized by *dru* typing. The characteristics of the majority of the isolates indicated a human origin, rather than an association with livestock. The results further revealed a considerable heterogeneity among the MRSA isolates, despite their common origin. Overall, a plenitude of major virulence factors and antimicrobial resistances was detected among the isolates, highlighting the potential risks associated with contaminated meat products and the transportation of such products among different countries.

## 1. Introduction

Transmission of *Staphylococcus* (*S*.) *aureus*, a pathogen which can cause a wide variety of diseases ranging from mild skin infections to severe conditions such as toxic shock syndrome and necrotizing pneumonia [1], occurs primarily through skin-to-skin contact, but contaminated objects and surfaces can also be the source of infections [2]. Foodstuffs, particularly products of animal origin, can serve as a vector, usually as a result of (cross-)contamination during handling in the course of production [3]. Furthermore, *S*. *aureus* can produce heat-stable enterotoxins, which can lead to symptoms such as nausea, diarrhea or abdominal cramping even after the respective bacteria have been destroyed by cooking [4].

Considering the extent of international trade with products of animal origin, distribution of pathogens such as *S*. *aureus* via these trade goods constitutes the potential for global dissemination. The European Union (EU) strives for a high level of food safety in order to ensure consumer health and, thus, a range of directives and regulations regarding the import of food were established. Microbiological criteria concerning meat products as well as all related imports to the EU were defined in Regulation (EC) 2073/2005 while Regulation (EC) No 206/2009 lays down strict rules and measures for the illegal import of products of animal origin in personal consignments [5]. Nevertheless, substantial amounts of meat products are introduced illegally into the EU each year, circumventing any controls. They are often carried in air passenger luggage, uncooled and sometimes over extended periods of time. This is especially worrying in the case of products originating from non-EU countries, where production hygiene and surveillance frequently do not meet EU standards and legal requirements. Furthermore, many of these products are home-made where the conditions of food processing are unknown [6].

In this study, we investigated *S*. *aureus* isolates recovered from meat and meat products confiscated from passengers returning from non-EU countries at two German airports and from sea freighted samples of legally imported meats from non-EU countries.

## 2. Materials and methods

### 2.1. Collection of bacterial isolates and molecular analyses

The isolates used in this study originated from meat and meat products introduced to Germany from January 2014 through January 2015, both illegally and legally. The analysed samples of illegally imported meat products were taken from confiscates seized during routine passenger controls at Berlin Schönefeld Airport (SXF) and Frankfurt International Airport (FRA). These samples comprised poultry meat (n = 43) as well as pork meat and products thereof (n = 108). Samples of legally imported poultry meat (n = 231) were collected at the border inspection post Hamburg port. All samples were stored on-site in separate sterile bags, marked and frozen by the competent authorities. Subsequently, the frozen samples were transferred in batches to the Institute of Food Quality and Food Safety, Hannover for testing. Additionally, commercially available, frozen, boneless pork filets (n = 66), of Chilean origin were examined, representing legally imported pork meat. They were purchased on 3 occasions in Lower Saxony, in a store of a major German cash-and-carry wholesaler with international distribution. Preparation of the samples was performed according to ISO 6887–2:2003, followed by detection and enumeration of *Staphylococcus* spp. according to DIN EN ISO 6888–1. ChromID MRSA Agar (Biomerieux, Marcy-l’Etoile, France) was used to screen for methicillin/oxacillin resistance. All presumptive methicillin-resistant *S*. *aureus* as well as an assortment of methicillin-sensitive isolates (MSSA) were examined further.

Total genomic DNA was obtained from overnight cultures by using the DNeasy Blood & Tissue Kit (QIAGEN, Hilden, Germany) according to the manufacturer’s instructions. A specific PCR assay targeting the *nuc* gene, was used to identify *S*. *aureus* isolates [7]. Methicillin resistance was confirmed via PCR amplification and subsequent gel electrophoresis of internal fragments of the *mecA* and *mecC* genes as described previously [8, 9]. Primers forward (5'-GGAAACTTGGGATAG-3’) and reverse (5'-AACCATCACATACCG-3’) were used to amplify an internal fragment of the *cat(pSBK203R)* gene and, in addition to previously described primers [10], *fexA* forward (5'-GTTAGTTTGGAGAACCG-3’) and reverse (5'-CCACTTGCCAGAATC-3’) were used to amplify an internal segment of the *fexA* gene. Sequencing of the transpeptidase domain of *pbp2* was performed using previously described primers [11]. Primer pairs *pbp1* forward (5'-GAGAAGCAGCCTAAACG-3’) and reverse (5'-TTGATTGGTGGGACAC-3’) as well as *pbp3* forward (5'-GCAGCAGTTTCTCAGC-3’) and reverse (5'-CATCTCTACCTAAGTCTCCAC-3’) were used for amplification and sequencing of internal fragments of *pbp1* and *pbp3* respectively.

Microarray analysis was performed using the *S*. *aureus* Genotyping Kit 2.0 (Alere Technologies, Jena, Germany) following the manufacturer’s instructions. It further allowed the assignment to clonal complexes and known lineages based on hybridization patterns. Detailed information on the procedure, probes and target genes have been published previously [1, 12]. In addition, the presence of the resistance determinants *tet*(L) and *erm*(T), as well as the avian-associated mobile genetic elements, which were not yet included in the microarray, was investigated by PCR as described previously [13–15].

*Spa* typing was performed as described by Harmsen et al. [16]. Sequencing of purified amplicons was performed by Eurofins Genomics (Eurofins Genomics, Ebersberg, Germany). Of each *spa* type detected, one isolate was further analyzed by multilocus sequence typing (MLST) according to Enright et al. to confirm clonal complex assignments [17]. For methicillin-resistant *S*. *aureus* (MRSA) isolates, the number of SCC*mec*-associated direct repeat units (*dru*) was determined as described previously [18]. *Dru* types were assigned using the data available at http://dru-typing.org.

Macrorestriction analysis was performed according to the HARMONY protocol [19]. The BioNumerics software (Applied Maths, Sind Martens-Latem, Belgium) was used for band pattern analysis using the Dice coefficient with 0.5% optimization and 1% position tolerance.

### 2.2. Antimicrobial susceptibility testing and β-lactamase inhibition test

Minimum inhibitory concentrations (MICs) for the following 22 antibiotics/antibiotic combinations were determined via broth microdilution method using customized microtiter plates (Sensititre, East Grinstead, UK): amoxicillin/clavulanic acid, ampicillin, cefazolin, cefoperazone, cefotaxime, cefquinome, ceftiofur, ciprofloxacin, clindamycin, enrofloxacin, erythromycin, gentamicin, lincomycin, marbofloxacin, oxacillin, penicillin, pirlimycin, tetracyclin, tilmicosin, trimethoprim/ sulfamethoxazole, tylosin and vancomycin. In addition, MIC values for some antibiotics (chloramphenicol, daptomycin, linezolid, spiramycin, florfenicol, kanamycin, and trimethoprim), which were not included in the microtiter plate layouts, were determined by broth macrodilution susceptibility testing. The tests were performed and interpreted (where applicable) in accordance with the Clinical and Laboratory Standards Institute (CLSI) guidelines and *S*. *aureus* ATCC 29213 was used for quality control purposes [20–23].

Strains not carrying the *mecA* or *mecC* gene but exhibiting low-level oxacillin resistance were further investigated by β-lactamase inhibition testing. In brief, oxacillin MICs were determined in the presence and absence of clavulanic acid and results were compared. Testing was carried out by broth microdilution in cation-adjusted Mueller-Hinton broth supplemented with 2% NaCl in a twofold dilution series. MICs were determined in parallel by adding equal concentrations of oxacillin and clavulanic acid (1:1 ratio) and by adding a fixed concentration of 4 μg/ml clavulanic acid in addition to oxacillin [24].

### 2.3. Nucleotide sequence accession numbers

The nucleotide sequences of *fexA* obtained from isolate 333 and the sequence of *pbp2* from isolate 632 have been deposited in GenBank under accession numbers KX230476 (*fexA*) and KX230475 (*pbp2*).

## 3. Results

### 3.1. MRSA and MSSA isolates and their origins

Based on the detection of the *mecA* gene, nine isolates could be classified as MRSA. Another 5 isolates also showed resistance to oxacillin but were tested negative for *mecA* and *mecC*. In the following, however, MRSA refers only to the oxacillin-resistant isolates carrying *mecA*.

All MRSA isolates originated from Egypt and were found in illegally introduced poultry meat seized at SXF. No MRSA were found in samples taken at FRA or in samples from legally imported meats. In addition, 14 out of 65 MSSA isolates were examined further for comparison reasons. These isolates were selected so as to comprise legally and illegally imported samples of both poultry and pork, originating from 8 different non-EU countries and including only one isolate per sample (Table 1). In order to reflect the common origin of all detected MRSA isolates, a higher number of MSSA isolates from separate poultry samples of Egyptian origin was included in the study.

**Table 1:** Origin of *S*. *aureus* isolates included in the study.

Isolate number	Sampling location	Date*	Origin	Legal status	Matrix	MRSA
181	Berlin Schönefeld	2015 Jan 3	Egypt	illegal	poussin	+
333	Berlin Schönefeld	2014 Dec 9	Egypt	illegal	whole chicken	+
334	Berlin Schönefeld	2014 Dec 9	Egypt	illegal	whole chicken	+
478	Berlin Schönefeld	2014 Feb 21	Egypt	illegal	whole chicken	+
649	Berlin Schönefeld	2014 Sep 27	Egypt	illegal	whole chicken	+
651	Berlin Schönefeld	2014 Sep 27	Egypt	illegal	whole chicken	+
652	Berlin Schönefeld	2014 Sep 27	Egypt	illegal	whole chicken	+
655	Berlin Schönefeld	2014 Sep 27	Egypt	illegal	cut duck	+
667	Berlin Schönefeld	2014 Sep 27	Egypt	illegal	cut duck	+
284	Hamburg	2014 Apr 23	Brazil	legal	salted chicken meat	-
316	Hamburg	2014 May 6	Argentina	legal	chicken meat	-
341	Berlin Schönefeld	2014 Dec 28	Egypt	illegal	whole duck	-
430	Berlin Schönefeld	2014 Jan 26	Vietnam	illegal	blood sausage	-
534	Berlin Schönefeld	2014 May 2	Russia	illegal	raw sausage	-
535	Berlin Schönefeld	2014 May 15	Belarus	illegal	raw sausage	-
585	Hamburg	2014 Oct 20	Thailand	legal	salted chicken meat	-
632	Hamburg	2014 Nov 4	Chile	legal	chicken meat	-
635	Hamburg	2014 Nov 4	Brazil	legal	chicken meat	-
641	Berlin Schönefeld	2014 Sep 6	Egypt	illegal	poussin	-
642	Berlin Schönefeld	2014 Sep 6	Egypt	illegal	poussin	-
643	Berlin Schönefeld	2014 Sep 6	Egypt	illegal	poussin	-
647	Berlin Schönefeld	2014 Sep 6	Egypt	illegal	poussin	-
693	Wholesaler	2014 Nov 25	Chile	legal	pork filet	-

*Date of sample acquisition, if unknown: date of first isolation

*Spa* typing revealed 10 different *spa* types, most prevalent was t127. The remaining isolates belonged to t376 (n = 3), t002 (n = 2), t084 (n = 2), t105 (n = 1), t131 (n = 1), t267 (n = 1), t318 (n = 1), t688 (n = 1) or t786 (n = 1). Each *spa* type correlated with a different MLST sequence type, with the exception of t105 and t688, which were both associated with ST5. Overall, 9 different MLST sequence types were detected. Three of these were novel sequence types and were assigned to ST3216 (CC15), ST3217 (CC80) and ST3218 (CC5). Derived from the results of MLST and the microarray analysis, most MSSA isolates belonged to CC1 (n = 8), which was also the case for 2 MRSA isolates. CC5 was represented by 3 MSSA and 1 MRSA isolate while another two MSSA belonged to CC15. The remaining MSSA isolate was associated with CC7 according to the results of the microarray analysis but could ultimately be classified as a singleton (t131/ST1290). Three MRSA isolates were assigned to CC80, while CC30, CC88, and CC97 were present with single MRSA isolates each. *Dru* typing revealed dt10a as the most prevalent *dru* type, which was present in 4 isolates. Two novel *dru* types were discovered among 3 isolates, designated dt11dc and dt10dg. Typing of the staphylococcal cassette chromosome *mec* (SCC*mec*) of the MRSA isolates based on the results of the microarray analysis, revealed SCC*mec* IV in 5 isolates and SCC*mec* V in 3 isolates. One isolate was non-typable harboring a classC *mec* element accompanied by *fusC* and *ccrAB*1 recombinase genes.

**Fig 1:**
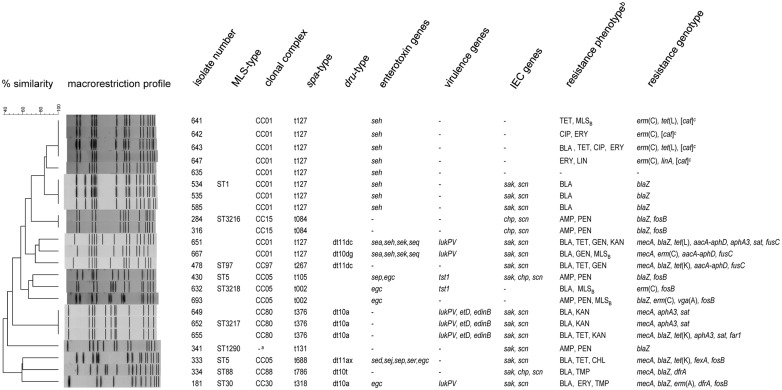
Genetic relatedness, major virulence factors, antimicrobial resistance phenotypes and genotypes detected in the 23 isolates. Isolates displaying resistance to penicillin, ampicillin and oxacillin are considered resistant to β-lactam antibiotics. For other isolates, resistance to individual β-lactam antibiotics is specified. ^a^ Singleton ^b^AMP = ampicillin; BLA = β-lactams (oxacillin-resistant isolates are considered resistant to all β-lactam antibiotics, except for those with anti-MRSA activity, according to CLSI document M100-S24); CHL = chloramphenicol; CIP = ciprofloxacin; ERY = erythromycin; GEN = gentamicin; KAN = kanamycin; LIN = lincomycin; MLS_B_ = macrolides, lincosamides, streptogramin B; OXA = oxacillin; PEN = penicillin; TET = tetracycline; TMP = trimethoprim ^c^gene detected by microarray analysis but could not be confirmed by PCR and does not confer phenotypical resistance

Results correlated well with macrorestriction profiles obtained for the isolates. Isolates with the same *spa*- and MLST sequence type showed up to 100% similarity in band patterns. CC1 and CC5 isolates can be divided into two clusters each. In both cases one cluster comprises the MSSA isolates while the other includes the MRSA isolates. The CC1 MSSA can be further divided into 2 sub-clusters, correlating with different resistance gene patterns and the presence or absence of genes belonging to the immune evasion cluster, respectively. Overall, the results were very heterogeneous, reflecting a considerable diversity among the isolates (Fig 1).

### 3.2. Microarray analysis and detection of virulence genes

Microarray analysis revealed a number of different virulence genes (Fig 1). The Panton-Valentine leukocidin (PVL) encoding genes could be detected in 6 isolates, all MRSA. Fifteen isolates were positive for at least one staphylococcal enterotoxin (SE) gene. Two isolates carried *sea*, in both cases in combination with PVL. Moreover, all t127 isolates harbored *seh*, 2 of which also tested positive for PVL. Further enterotoxin genes detected include *sed* (n = 1), *sej* (n = 1), *sek* (n = 2), *sep* (n = 2), *seq* (n = 2), *ser* (n = 1), and the enterotoxin gene cluster (egc) which was present in 5 isolates. Furthermore, 3 CC80 MRSA isolates carried *etD*, coding for exfoliative toxin D, and *edinB*, encoding epidermal cell differentiation inhibitor B. The toxic shock syndrome toxin gene (*tst1*) was detected in 2 CC5 MSSA isolates. Furthermore, 16 isolates carried 2 or 3 genes belonging to the immune evasion cluster (IEC): *sak*, *chp*, and *scn*, encoding staphylokinase, a chemotaxis inhibiting protein, and a staphylococcal complement inhibitor, respectively.

PCR assays targeting avian-associated mobile genetic elements detected φAvβ and pAvX in one CC5 isolate and pAvY in another CC5 isolate.

*Agr* typing revealed *agr* type III as the dominant type, which was present in 15 isolates. The other isolates either carried allele type II (n = 6) or type I (n = 2). All of the *agr* type III isolates were of capsule type 8 (*cap 8*), as were two *agr* type II isolates and one *agr* type I isolate. The remaining isolates belonged to capsule type 5 (*cap 5*).

### 3.3. Antimicrobial resistance phenotypes and genotypes

Antimicrobial resistance patterns and the according genotypes are displayed in Fig 1. Most commonly observed was resistance to β-lactam antibiotics. In 5 cases, the resistance was limited to penicillin and ampicillin, whereas the other isolates were additionally resistant to oxacillin. Interestingly, 6 isolates displayed elevated MICs for oxacillin despite their lack of the *mecA* and *mecC* genes. Five of these had MIC values above the breakpoint for oxacillin resistance (MICs of 4 to 8 μg/ml) while 1 isolate displayed a MIC one dilution step below the breakpoint (MIC of 2 μg/ml). When tested for increased β-lactamase production by inhibiting β-lactamase activity with clavulanic acid, MICs for oxacillin either stayed the same or were lowered only by 1 twofold dilution step. In fact, 3 of the isolates did not even carry the *blaZ* gene. Furthermore, internal fragments of the respective *pbp1*, *pbp2* and *pbp3* genes were sequenced and compared with the nucleotide sequence from *S*. *aureus* NCTC 8325 deposited in the databases (GenBank accession no CP000253.1) [11]. All 6 isolates displayed a Thr438→Ser substitution in the *pbp3* gene and all isolates except isolate 632 exhibited a Val625→Met substitution in *pbp1*. These sequences, however, matched previously published sequences of other MSSA (e.g. accession nos. BX571857.1, CP015646.1). The *pbp2* gene of one isolate, isolate 632, exhibited a mutation resulting in an Asn598→Lys amino acid substitution.

Two isolates showed low-level resistance to ciprofloxacin (MIC values of 4 and 8 μg/ml). In both cases, the MIC value for enrofloxacin was 1 μg/ml and 2 μg/ml for marbofloxacin. Sequencing of the *grlA*, *grlB*, *gyrA*, and *gyrB* genes revealed a mutation in the *grlA* gene of both isolates when compared with wild-type *grlA* [25], resulting in a Ser80→Phe amino acid substitution. Resistance determinants *erm*(C) or *erm*(A), conferring resistance to macrolides, lincosamides and streptogramin B (MLS_B_) antibiotics were found in 8 isolates. One isolate was also positive for *vga*(A) in addition to *erm*(C). All of these isolates were phenotypically resistant to erythromycin with MICs of >32 μg/ml. Three isolates also showed resistance to clindamycin and elevated MIC values to further MLS_B_ antibiotics tested in this study (pirlimycin, spiramycin, tilmicosin, tylosin, lincomycin). One isolate displayed an elevated MIC value only for lincomycin in addition to phenotypical resistance to erythromycin. This isolate carried *lin*(A) in addition to *erm*(C).

Three isolates displayed resistance to gentamicin, which was mediated by the *aacA-aphD* genes in all cases. Tetracycline resistance, which was present in 6 isolates, was mediated by *tet(*K) or *tet*(L).

In 4 isolates which were classified as phenotypically susceptible to chloramphenicol (MICs of 2 to 8 μg/ml), microarray analysis revealed the presence of the chloramphenicol resistance gene *cat(pSBK203R)*. However, the gene *cat(pSBK203R)* could not be confirmed by using a specific PCR assay. Only 1 isolate showed phenotypic resistance to chloramphenicol (MIC of >64 μg/ml) but not elevated MICs to florfenicol and harbored *fexA*. Since *fexA* usually confers combined resistance, internal fragments of the gene were amplified by PCR, sequenced and compared with the sequence originally detected in *S*. *lentus* (GenBank accession no. AJ549214.1). Compared with the previously published sequence, 4 sequence alterations leading to amino acid substitutions Gly33→Ala, Ala37→Val, Ile131→Val, and Pro321→Thr were detected in isolate 333.

With regard to genes conferring elevated MICs to folate pathway inhibitors, 2 isolates were tested positive for *dfrA*. Even though these isolates were not classified as resistant to the combination of trimethoprim/sulfamethoxazole (1:19), they had MICs of ≥128 μg/ml trimethoprim which is above the clinical breakpoint for trimethoprim resistance (≥16 μg/ml). Kanamycin MICs were determined for the 4 isolates carrying *aphA3*. Those isolates all proved to be resistant to kanamycin with MICs of ≥256 μg/ml. The gene *fosB* was detected in 7 isolates. When tested for fosfomycin susceptibility via Etest, 6 isolates exhibited MICs of 2–3 μg/ml and the remaining isolate showed a slightly higher value of 6 μg/ml.

Even though phenotypical resistance to the according substances was not determined in the course of this study, further resistance genes were detected through microarray analysis including the unspecific efflux pump gene *tetEfflux*, which was present in all isolates, the genes *far1* (n = 1) and *fusC(Q6GD50)* (n = 4), conferring resistance to fusidic acid, and the gene *sat* (n = 4), conferring resistance to streptothricin.

No isolate displayed resistance to vancomycin, daptomycin, linezolid or the combination sulfamethoxazole/trimethoprim.

## 4. Discussion

Since the beginning of air travel, passenger numbers have constantly been increasing. In 2011, the International Air Transport Association (IATA) reported a total of 2.8 billion passengers, 1.1 billion of which were international travelers. In 2014, the total passenger number increased to 3.3 billion and is expected to reach 7 billion in 2034. African, Asian, and South American countries are expected to show a particularly fast growth [26, 27]. As a result, the total amount of products of animal origin carried in passenger luggage, including meat products, can be expected to rise accordingly. As passengers are only spot-checked, the minority of illegally imported meat products are discovered. Nonetheless, meat products seized at SXF and FRA in 2014 amounted to 1.2 tons and 1.6 tons, respectively [28]. Therefore, it is increasingly important to assess the risk of global dissemination of pathogens and antimicrobial resistances via this route. Virtually all of the isolates used in this study originated from countries in the aforementioned regions. In particular, all *mecA*-positive isolates were of Egyptian origin, where a high contamination rate with MRSA has been reported for retail chicken meat, possibly due to insufficient hygiene during handling as well as ample use of antimicrobial agents [29]. Notably, many of our samples appeared to be home-made, where adherence to hygienic standards during slaughter and handling is likely to be inferior when compared to commercially produced meat. It should also be noted that Egypt is not included in the list of countries from which poultry meat may be imported into the EU, laid down in EC 798/2008, so there are no legal poultry imports of Egyptian origin for comparison. This could explain why all MRSA found in this study were isolated from illegally introduced meat. Instead, legal poultry samples were predominantly of South American origin. In 2014, more than 830 tons of poultry meat were imported into the EU, the majority (approx. 60%) from Brazil [30]. Consequently, legal poultry samples in this study were mostly of Brazilian origin. In Brazil, *S*. *aureus* has been reported as the most common cause of bloodstream and soft tissue infections in hospitalized patients, 31% being MRSA [31]. Overall, a difference in MRSA prevalence in the countries of origin should be considered a potential factor, rather than the legal status of the meats alone.

Despite their common origin, the MRSA isolates showed a marked diversity. The *mecA*-negative isolates on the other hand, while from various countries of origin and isolated from different matrices, were less divergent. Most isolates belonged to CC1 or CC5, which are common clonal complexes found in humans and animals [1]. Animal-associated lineages are usually negative for genes of the IEC, which was true for 5 of the 10 CC1 isolates and 2 out of 4 CC5 isolates, suggesting an animal origin in those cases. This is also reflected by the results of the macrorestriction analysis, leading to separate clusters of isolates carrying genes of the IEC and isolates without those genes, respectively. CC5 strains in particular are frequently isolated from chicken and also found in other livestock [32]. Isolates of avian origin have been described to carry specific mobile genetic elements comprising φAvβ, φAv1, SaPIAv, pAvX and pAvY [15]. One isolate, isolate 632 did carry φAvβ and pAvX, suggesting an adaptation to avian hosts. Another isolate, isolate 430, tested positive for pAvY only. This isolate also tested positive for 3 genes of the IEC, however, and it was isolated from a pork sample rather than from poultry. This suggests that pAvY might sometimes be present in isolates not associated with poultry. Instead of ST5, one of our CC5 isolates belonged to ST3218, a single locus variant of ST5.

No isolate belonged to CC398, which, in Western Europe, is the predominant CC associated with livestock-related strains. Overall, the majority of the isolates and the MRSA isolates in particular appear to be of human origin rather than livestock-associated.

PVL-positive CC80-MRSA harboring SCC*mec* IV are also known as the European CA-MRSA clone. Despite this designation, they are widely distributed in the Middle East, including Egypt. The 3 CC80-MRSA-IV isolates identified in the course of the current study all carried *aphA3* and *sat* in addition to PVL, which is characteristic for this strain, as well as *etD + edinB* [1, 33]. Overall, a number of different virulence genes were detected among the isolates in this study. Three isolates carried either *sea* or *sed*, and *seh* was present in the 10 CC1 isolates. In addition to their ability to cause food poisoning, SEs are staphylococcal superantigens (SSA). Consequently, infections with SSA producing strains can lead to severe systemic conditions, which are potentially fatal [34]. This is also true for the toxic shock syndrome toxin encoded by *tst1*, which was identified in 2 of our MSSA isolates. Since the vast majority of the samples used in this study consisted of uncooked meat, especially poultry, it can be assumed that heat treatment would have occurred before consumption. Nevertheless, preformed SEs, (cross-)contamination of surfaces and kitchen appliances with *S*. *aureus* as well as potential colonization or infection of persons handling the meat prior to cooking remain a risk and dissemination might occur [35].

In addition, the isolates in the current study displayed a variety of antimicrobial resistances. Overall, 14 isolates can be considered multi-resistant due to a genotype conferring resistance to three or more classes of antimicrobial agents [36]. This accounts for 8 out of 9 MRSA isolates and 6 out of 14 of the remaining isolates. In contrast, a study conducted on various products of animal origin illegally introduced to Spain revealed no further antibiotic resistances in addition to those against β-lactams in the detected MRSA and around 50% of the examined *S*. *aureus* isolates did not display any antimicrobial resistances [37]. Among the isolates carrying *erm*(A) or *erm*(C), constitutive as well as presumably inducible expression could be observed [38]. The *fosB* gene, encoding a Mn2+-dependent fosfomycin-inactivating enzyme, was detected by microarray analysis in seven isolates. Currently, there are no CLSI approved fosfomycin breakpoints available for *S*. *aureus*, but based on EUCAST guidelines all isolates in this study would be considered susceptible (MIC ≤32 μg/ml). The same has been described for *S*. *aureus* isolates in a previous study by Meemken et al [39]. For now, the reason for the functional inactivity of *fosB* in our isolates remains unclear.

In addition to various antimicrobial resistance genes, we identified mutations in topoisomerase genes which influence susceptibility to quinolones. The Ser80→Phe amino acid substitution detected in the deduced GrlA protein of 2 isolates was previously described to play a major role in quinolone resistance in *S*. *aureus*, although high MIC values were only observed in the presence of additional mutations in *grlA*, *grlB* or *gyrA*, which is in agreement with our results [40]. Another MRSA isolate carried a variant of *fexA*, which confers resistance to chloramphenicol but not elevated MICs of florfenicol, presumably as a result of amino acid substitutions Gly33→Ala and Ala37→Val as described by Gómez-Sanz et al. [41]. It further carried an exchange, leading to a Pro321→Thr substitution, which was not detected in the previously characterized *fexA* variant. Another interesting observation was the low-level oxacillin resistance in several isolates lacking *mecA*. None of our isolates showed a considerable reduction of oxacillin MICs under the influence of the β-lactamase inhibitor clavulanic acid. Several isolates were *blaZ* negative and for the remaining isolates, excessive β-lactamase production does not appear to be responsible for the low–level oxacillin resistant phenotype. However, the presence of a plasmid-mediated methicillinase, which has been discussed as one possible mechanism responsible for this phenotype, remains a possibility [42]. The Asn598→Lys substitution in the transpeptidase domain of *pbp2*, which is located in proximity to several previously identified amino acid substitutions in low-level oxacillin resistant isolates might explain the reduced oxacillin susceptibility in one isolate [11].

Even though the number of isolates analyzed in this study was relatively small, the plenitude of virulence factors and antimicrobial resistances in the isolates underline the potential risk associated with the import of meat and meat products contaminated with *S*. *aureus* into the EU. Multi-resistant strains in particular can contribute to the distribution of resistance genes and pose a threat to the consumer. Thus, transmission of *S*. *aureus* via food items, particularly through illegally conveyed products, should not be disregarded.
